# Adhesiveless Bonding of Metal Buttons to Clear Aligners: A Technical Report

**DOI:** 10.7759/cureus.88937

**Published:** 2025-07-28

**Authors:** Viet Anh Nguyen

**Affiliations:** 1 Faculty of Dentistry, Phenikaa University, Hanoi, VNM

**Keywords:** adhesiveless bonding, clear aligner therapy, elastic traction, metal orthodontic buttons, orthodontic auxiliaries

## Abstract

Auxiliary buttons are often required in clear aligner therapy for elastic traction. We introduce an adhesiveless flame-fusion technique for affixing auxiliary metal buttons to clear aligners, aiming to eliminate the extra chair time, occasional debonding, and translucent haze associated with resin adhesives. Round‑base stainless‑steel buttons are grasped with locking forceps, heated in an alcohol flame (~600-650 °C for 10-15 s), and immediately pressed with moderate manual pressure (~5 N) for 5-10 s onto 0.75 mm polyethylene terephthalate glycol (PETG) aligners that are fully seated on working models to prevent distortion. Clinical use has shown the buttons remain firmly embedded under routine intra‑ and inter‑arch elastic forces without compromising aligner fit or transparency, while bonding can be completed in well under a minute. This simple, cost-effective protocol is readily adaptable to most commercial aligner systems and avoids the drawbacks of adhesive bonding, including extra chair time, occasional debonding, and the translucent haze associated with resin adhesives.

## Introduction

Clear aligner therapy has become increasingly popular due to its aesthetic advantages and patient comfort; however, certain orthodontic movements, such as space closure, canine traction, posterior intrusion, and intermaxillary corrections, often necessitate the use of auxiliaries, including buttons, hooks, or precision-cut attachments, to anchor elastics and deliver targeted forces [1]. Clinical trials and systematic reviews have shown that the integration of aligner auxiliaries significantly enhances the efficiency and predictability of complex tooth movements compared to aligners alone [2,3]. Finite element analyses further demonstrate that precision-cut hooks and lingual buttons can distribute forces more evenly across the arch, improving anchorage control and minimizing unwanted side effects [4].

Hooks incorporated directly into the aligner allow for straightforward elastic attachment, facilitating effective traction in cases of impacted canine exposure and Class III malocclusion correction [5]. Despite these benefits, conventional bonding methods require adhesives, such as proprietary aligner adhesives, or precision cuts, which add chairside time, introduce inventory management challenges, carry a risk of premature detachment under elastic forces, and can impair aligner aesthetics by reducing transparency [6].

To address these limitations, we propose a novel adhesiveless bonding technique that leverages thermal softening of the thermoplastic and mechanical engagement to securely embed metal buttons onto clear aligners. By heating the stainless-steel button, heat is conducted into the adjacent aligner polymer, causing localized softening that allows the button flange to partially sink into the material. Upon cooling, the polymer re-solidifies around the button, mechanically interlocking it without any adhesive. This streamlined protocol preserves aligner fit and clarity while withstanding routine intra- and inter-arch elastic forces. This streamlined protocol eliminates adhesive handling and preserves aligner fit and clarity while withstanding routine intra- and inter-arch elastic forces.

## Technical report

**Figure 1:**
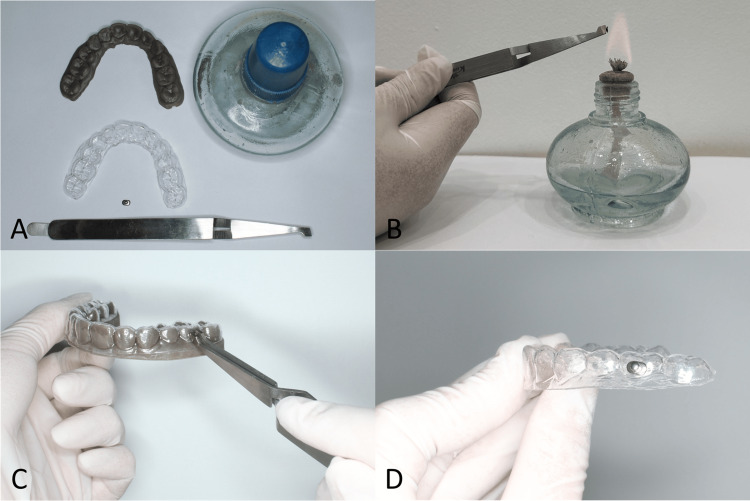
Adhesiveless bonding of a metal button to a clear aligner. (A) Setup; (B) button heated; (C) button pressed onto aligner; (D) final bonded appliance.

The armamentarium for this adhesiveless bonding technique consists of a working model of the patient’s dentition, the clear aligner, a prefabricated stainless-steel orthodontic button in any shape (IA04-051, 3B, Hangzhou, China), a bracket-holding instrument (HI34-001, 3B, Hangzhou, China) or tweezers with fine, non-serrated tips, and an alcohol burner or small butane torch (Figure 1A). Aligner materials can be polyethylene terephthalate glycol (PETG), polyurethane (PU), copolyester, or 3D-printed materials, with thickness ranging from 0.5 to 0.7 mm. To begin, seat the aligner firmly over the model to support its intaglio surface and prevent distortion when heat is applied. Position the working model on a stable, heat-resistant surface, such as a granite countertop. Next, grasp the metal button with the bracket holder and gently preheat its convex surface by inserting it directly into the burner flame at 600-650°C for 10-15 s just until it feels warm to the touch, not glowing red (Figure 1B). Flux is not necessary. Immediately transfer and press the heated button onto the outer surface of the aligner at the predetermined attachment site, applying consistent moderate finger pressure of approximately 5 N for 5-10 s to allow the thermoplastic to soften and conform around the button’s base (Figure 1C). Once the metal has cooled to room temperature, typically within 20-30 s, the button will be securely embedded in the aligner material. Carefully remove the aligner from the model (Figure 1D) and inspect the bond interface under 5× magnification with loupes to confirm uniform adaptation and absence of gaps. Typically, the metal button embeds into the aligner by about one-third of its thickness, with no voids observed.

## Discussion

The adhesiveless bonding technique presented here provides a streamlined, adhesive-free alternative for securing metal buttons to clear aligners. Our clinical observations that heated buttons mechanically engage the softened thermoplastic without debonding under routine intra- and inter-arch elastic forces align with the shear bond strength ranges reported by Pariyatdulapak et al., who demonstrated that PETG-based aligner materials achieved higher bond strengths than PU substrates and that most failures occurred at the adhesive-substrate interface [6]. By eliminating adhesives, our method removes the interface prone to failure while maintaining comparable retention on PETG sheets.

Finite element analysis by Jia et al. demonstrated that lingual buttons and precision cuts distribute orthodontic forces evenly, thereby improving anchorage control [3]. Although our protocol does not employ precision cuts, the locally softened polymer conforms tightly around the button base to create a mechanical undercut, achieving equivalent force transfer without the need for complex attachment designs. Moreover, the network meta-analysis by Alam et al. confirmed that the use of auxiliary attachments significantly enhances the accuracy and predictability of complex tooth movements compared to aligners alone [2]. Our adhesiveless method meets emergent elastic traction requirements chairside, without additional adhesives or inventory management, thus improving clinical efficiency and reducing overall treatment time. Finally, Li et al. emphasized the importance of preserving the mechanical properties and transparency of thermoplastic aligners when integrating auxiliaries [4]. Our preliminary observations, based on visual inspection and short-term use, indicate that brief, localized heating does not appreciably alter the bulk properties or clarity of the aligner material, in line with their recommendations for maintaining aligner performance.

Gange described the development of Bond Aligner, a specialty composite formulated to bond hooks or buttons directly to clear aligners [7]. Bond Aligner chemically bonds to all clear aligner thermoplastics and matches their modulus of elasticity, enabling attachments to flex in concert with the appliance without peeling or flaking; moreover, Bond Aligner can be applied to the occlusal surface to achieve bite-opening effects. However, to our knowledge, no study has described an adhesive-free method for bonding buttons to aligners. By eliminating adhesive handling, polymerization variables, and inventory requirements, our protocol reduces chairside complexity and potential sources of error, while preserving aligner fit and transparency.

A limitation of this study is the absence of quantitative bench testing under standardized conditions such as shear strength tests, thermocycling, and aging. Another important limitation is the lack of biocompatibility evaluation: heating the aligner material could alter its physical or chemical state and potentially leach harmful compounds, so future work must include cytotoxicity and chemical analyses to ensure safety. Future investigations should also compare this adhesiveless technique directly against Bond Aligner adhesive on both PETG and PU substrates, evaluating long‑term durability, fatigue resistance, and any effects on aligner mechanical properties. Nonetheless, for emergent elastic‑traction needs, especially when precision cuts were not incorporated into the initial treatment plan, this simple, cost‑effective method offers a reliable clinical solution.

## Conclusions

To our knowledge, this is the first reported adhesiveless thermal-bonding method for securing metal buttons to clear aligners. Short-term clinical observation has demonstrated reliable retention under routine elastic forces, with no discernible effect on aligner transparency, fit, or patient comfort. This streamlined protocol eliminates adhesive handling, reduces clinical steps, and preserves the aesthetic advantages of clear aligner therapy. The technique is particularly indicated for emergent elastic-traction needs, such as impacted canine exposure and Class III correction, and as a backup when precision cuts are absent, but may be contraindicated when using materials with low thermal resistance. While initial clinical use supports its efficacy, further in vivo and in vitro studies are needed to validate long-term durability, compare directly with conventional adhesive systems on PETG and PU substrates, evaluate potential changes in polymer integrity, and confirm biocompatibility after localized heating.

## References

[1] Kravitz ND, Kusnoto B (2007). A quick and inexpensive method for composite button fabrication. J Clin Orthod.

[2] Khursheed Alam M, Hajeer MY, Shqaidef A (2024). Impact of various aligner auxiliaries on orthodontic activity: A systematic review and network meta-analysis. Saudi Dent J.

[3] Jia L, Wang C, Li L (2023). The effects of lingual buttons, precision cuts, and patient-specific attachments during maxillary molar distalization with clear aligners: Comparison of finite element analysis. Am J Orthod Dentofacial Orthop.

[4] Li J, Si J, Xue C, Xu H (2024). Seeking orderness out of the orderless movements: An up-to-date review of the biomechanics in clear aligners. Prog Orthod.

[5] Vignolo Lobato R, Gonzalez Zamora D (2025). Innovative protocol for early class III correction with aligner and facemask: A case report. J Oral Med Dent Res.

[6] Pariyatdulapak N, Churnjitapirom P, Srikhirin T, Viwattanatipa N (2021). Bond strength of orthodontic buttons on clear aligner materials. Orthod Waves.

[7] Gange P (2017). An interview with Paul Gange. Dental Press J Orthod.

